# Brain Metastases from Breast Cancer Histologically Exhibit Solid Growth Pattern with at Least Focal Comedonecrosis: A Histopathologic Study on a Monocentric Series of 30 Cases

**DOI:** 10.3390/diagnostics13193141

**Published:** 2023-10-06

**Authors:** Jessica Farina, Giuseppe Angelico, Giada Maria Vecchio, Lucia Salvatorelli, Gaetano Magro, Lidia Puzzo, Andrea Palicelli, Magda Zanelli, Roberto Altieri, Francesco Certo, Saveria Spadola, Maurizio Zizzo, Giuseppe Maria Vincenzo Barbagallo, Rosario Caltabiano, Giuseppe Broggi

**Affiliations:** 1 Department of Medical and Surgical Sciences and Advanced Technologies “G.F. Ingrassia”, Anatomic Pathology, University of Catania, 95123 Catania, Italy; jessicafarina2693@gmail.com (J.F.); giuangel86@hotmail.it (G.A.); giadamariavecchio@gmail.com (G.M.V.); lucia.salvatorelli@unict.it (L.S.); g.magro@unict.it (G.M.); lipuzzo@unict.it (L.P.); rosario.caltabiano@unict.it (R.C.); giuseppe.broggi@phd.unict.it (G.B.); 2 Pathology Unit, Azienda USL-IRCCS di Reggio Emilia, 42123 Reggio Emilia, Italy; andrea.palicelli@ausl.re.it; 3 Department of Neurological Surgery, Policlinico “G. Rodolico-S. Marco” University Hospital, 95121 Catania, Italy; roberto.altieri.87@gmail.com (R.A.); cicciocerto@yahoo.it (F.C.); gbarbagallo@unict.it (G.M.V.B.); 4 Interdisciplinary Research Center on Brain Tumors Diagnosis and Treatment, University of Catania, 95123 Catania, Italy; 5 Pathology Unit, Cannizzaro Hospital, 95126 Catania, Italy; saveriaspadola@hotmail.it; 6 Surgical Oncology Unit, Azienda USL-IRCCS di Reggio Emilia, 42123 Reggio Emilia, Italy; maurizio.zizzo@ausl.re.it

**Keywords:** brain metastasis, breast cancer, diagnosis, comedonecrosis, solid growth pattern

## Abstract

Since there are no morphological clues capable of making a pathologist suspect a possible mammary origin of a metastatic lesion without adequate clinical information, the histologic diagnosis of brain metastasis from BC is still based on the immunohistochemical expression of mammary gland markers such as GATA-3, ERs, PgRs and HER-2. The present retrospective study aimed to select purely morphological features capable of suggesting the mammary origin of a metastatic carcinoma in the brain. The following histological features were collected from a series of 30 cases of brain metastases from breast cancer: (i) a solid growth pattern; (ii) the presence of comedonecrosis; and (iii) glandular differentiation. Our results showed that most cases histologically exhibited a solid growth pattern with at least focal comedonecrosis, producing an overall morphology closely reminiscent of mammary high-grade ductal carcinoma in situ. Although the above-mentioned morphological parameters are not strictly specific to a mammary origin, they may have an important diagnostic utility for leading pathologists to suspect a possible breast primary tumor and to include GATA-3, ERs, PgRs and HER-2 in the immunohistochemical panel.

## 1. Introduction

Brain metastases are the most common intracranial neoplasms in adults. The newly diagnosed brain metastasis incidence is 3 to 10 times higher than that of primary brain tumors [1]. In the last few decades, metastases in the central nervous system have gained increasing clinical interest due to the development of different therapeutic alternatives, such as surgical resection, whole-brain radiotherapy, radiosurgery and targeted systemic therapies, that have significantly improved the survival of these patients [1,2].

Specifically, given their greater frequency, breast and lung cancer are the most common tumors to metastasize to the brain [1,2], with the former developing in approximately 10–30% of the tumor population [1,3]. Intracerebral metastasis may represent the first manifestation of a systemic disease or, more commonly, present itself metachronously, with patients experiencing headaches, nausea, seizures, defects in speech, behavior, coordination and neurocognition, thus lowering their quality of life. Various risk factors have been identified and associated with the risk of brain metastatic breast cancer (BC), with one of the most recent systematic reviews including younger age, estrogen receptor (ER)-negative status, HER2-positive status, higher tumor stage, higher histologic grade, large tumor size and high Ki67 labelling index as independent risk factors [4,5]. Brain metastases have long been considered a late event in the progression of the disease, occurring even a decade after the primary cancer diagnosis, preceded by lung, liver and bone metastases, although it is not that uncommon to identify a direct link between BC and the brain metastasis course of the disease (≅12%) [4]. Some studies have tried to assess the microenvironment of brain metastases from BC, many of them suggesting a prognostic significance regarding the presence of necrosis, gliosis, immune infiltrate and hemorrhage [6].

The majority of patients affected by brain metastases from BC develop multiple intracerebral metastases, while a solitary mass occurs only in 14% of cases [1]; furthermore, BC frequently exhibits leptomeningeal spread via the hematogenous route, direct extension and/or extension along nerves and lymphatic vessels [1], and the pia madre or the arachnoid are the most commonly affected meningeal layers [1].

Since, as far as we are aware, there are currently no morphological clues capable of making a pathologist suspect a possible mammary origin of a metastatic lesion in the brain in the absence of adequate clinical information, the histologic diagnosis of brain metastasis from BC is mainly based on the immunohistochemical expression of mammary gland markers such as GATA-3, ERs, progesterone receptors (PgRs) and HER-2 [7].

The present retrospective study investigated the presence of potential recurrent histologic features in a series of 30 cases of brain metastases from breast carcinoma in order to collect some useful morphological features capable of suggesting to a pathologist a potential mammary origin of the neoplasm when clinical data are not available or the primary tumor is unknown.

## 2. Materials and Methods

The present research was carried out in accordance with the Declaration of Helsinki and obtained the approval of the local ethics committee, Catania 1 (CE 165/2015/PO). Histologic specimens of all brain metastases from BCs, surgically excised at the Neurosurgery Unit of the “Policlinico G. Rodolico-San Marco” University hospital between 2018 and 2023, were retrospectively collected. Surgical samples were fixed in 10% neutral buffered formalin, embedded in paraffin, cut to 4–5 microns and stained with hematoxylin and eosin; the corresponding clinical (age, gender and anatomic site of the metastatic tumor) and immunohistochemical data were retrieved from the original pathological reports. Hematoxylin- and eosin-stained sections were separately evaluated by two pathologists (J.F. and G.B.) who were blind to the clinical data of the patients. We collected the following histologic features: (i) solid growth pattern; (ii) presence of comedonecrosis; (iii) glandular differentiation. Their presence was graded by a semi-quantitative optical analysis according to a four-tiered system (0% of the tumor = absent; 1–10% of the tumor = focal; 11–50% of the tumor = heterogeneous; >50% of the tumor = diffuse), as previously described [8,9,10].

## 3. Results

The clinico-pathologic and immunohistochemical features of the cases from our series are summarized in Table 1.

### 3.1. Clinico-Pathologic Features

The study included 30 female patients (median age: 51.6 years; age range: 34–75). One patient had a further recurrence one year after their first brain metastasis diagnosis. Intracerebral metastases were found in 27 patients, while 3 patients had cerebellar involvement. Of those 27 patients with intraparenchymal metastasis, 6 had a temporal localization, 3 parietal, 3 frontal, 2 tentorial, 1 peri-trigonal, 1 pterional and 11 had no specific site reported on the original report.

**Figure 1:**
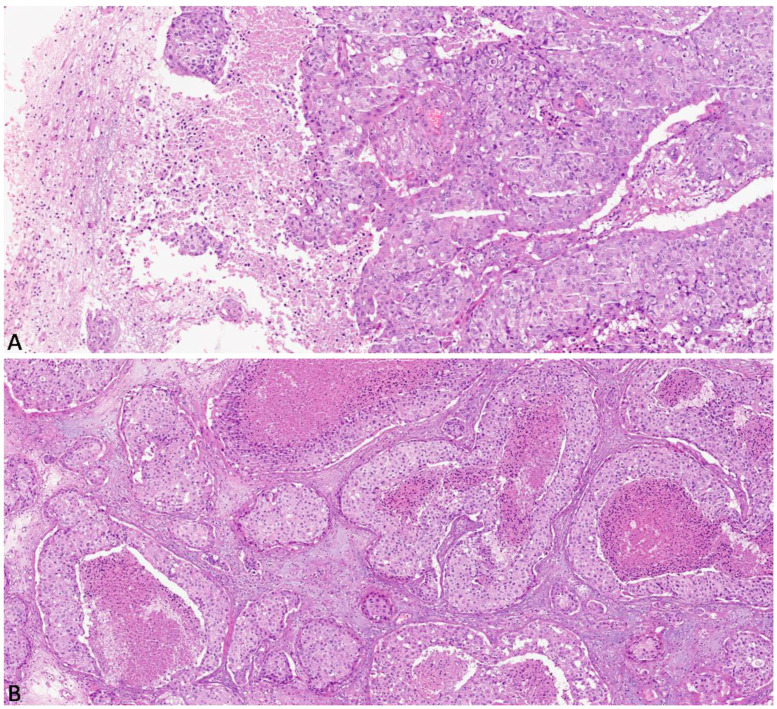
(**A**) Brain metastasis from breast cancer showing a diffuse solid growth pattern with no glandular differentiation (hematoxylin and eosin; original magnification 150×); (**B**) Diffuse solid growth pattern with comedonecrosis produces an overall morphology closely resembling a high-grade in situ ductal carcinoma of the breast (hematoxylin and eosin; original magnification 150×).

Histologically, 14 cases (47%) exhibited a diffuse solid growth pattern (Figure 1A), 9 cases (30%) had a heterogeneous solid growth pattern; the remaining 7 cases (23%) showed a focal solid growth pattern. Diffuse comedonecrosis was found in 7 cases (23%) (Figure 1B), while heterogeneous and focal comedonecrosis were found in 11 (37%) and 9 (30%) cases, respectively. Three cases (10%) showed no necrosis. Glandular differentiation was absent in 14 cases (47%), heterogeneous in 8 cases (27%) and focal in 8 cases (27%).

### 3.2. Immunohistochemical Features

Among the whole cohort of 30 cases of metastatic breast cancer that have spread to the brain, 8 cases (27%) showed a triple-negative/basal-like phenotype, 3 (10%) were luminal-A, 1 (3%) was luminal-B and 5 tumors (17%) were HER-2-enriched. In 13 cases (43%), the immunophenotype was not available in the original pathology report. The immunophenotypes of brain metastases were compared with those of primary breast tumors, and no discrepancies were found.

## 4. Discussion

Brain metastases from BC significantly impact patients’ quality of life [1,2,3]. BC is one of the most common types of cancer in women, and it has a high potential to spread to other organs, including the brain [11,12,13]. Several symptoms, including headaches, seizures, confusion, and difficulty with movement and coordination, may be caused by brain metastases [14,15,16]. The incidence of brain metastases from BC has been increasing in recent years, likely due to improvements in cancer treatments that have led to longer survival times [14,15,16,17,18]. However, the prognosis for patients with brain metastases remains quite poor, and overall survival is mainly influenced by the molecular phenotype of breast cancer, with triple-negative/basal-like tumors having the worst outcomes and HER-2-enriched ones having the best.

Brain metastases from BC currently represent therapeutic challenges often needing a combined treatment approach that may include surgery, radiation therapy, chemotherapy and targeted therapy [17,18,19,20]. Surgery may be used to remove a single metastasis or to relieve pressure on the brain caused by mass effect and peritumoral edema; radiation therapy can be adopted to shrink tumors and relieve symptoms, while chemotherapy and targeted therapy may be performed to manage extracranial disease. Multimodal combined therapies capable of controlling both intra- and extra-cranial disease are often desirable to improve overall patient survival [17,18,19,20]. As early diagnosis and aggressive treatment are crucial for improving outcomes for patients with brain metastases from BC, regular monitoring and imaging tests are highly recommended to detect brain metastases early. Additionally, ongoing research is needed to develop new and more effective treatments for brain metastases from BC.

While it is relatively straightforward for a pathologist to distinguish between a metastatic carcinoma in the brain and a primary central nervous system neoplasm, it may be more challenging to hypothesize the potential origin of the tumor on the basis of morphology alone and, above all, in the absence of accurate anamnestic information from other clinicians. Therefore, immunohistochemistry (IHC) is a mandatory ancillary method for the identification of the origin of a metastatic carcinoma in the cerebral parenchyma, ensuring that a purely morphological diagnosis of “adenocarcinoma” or “squamous cell carcinoma” is not rendered [21]. However, although IHC is currently a widely accessible and widespread method in almost all pathology units, the frequent lack of anamnestic information about the patient’s previous neoplasms means that pathologists must use a wide immunohistochemical panel when the primary tumor is unknown, trying to cover all the potential sites of origin of the neoplasm, with often a significant waste of resources and time.

The present research aimed to identify some purely morphological features of metastatic carcinomas in the brain, which could potentially be useful to suggest to the pathologist a potential mammary origin of the neoplasm. In more detail, our results demonstrated that the majority of metastatic BCs from our cohort histologically exhibited an almost “pure” solid growth pattern with at least focal comedonecrosis, producing an overall morphology closely reminiscent of mammary high-grade ductal carcinoma in situ.

In our series, as expected, most cases exhibited an immunoprofile consistent with the triple-negative/basal-like and HER-2-enriched subtypes. The possibility of differences in receptor status and/or genomic profiling between brain metastases from BC and a primary tumor has been reported in the literature [5]. In particular, the loss of ER, PgR and HER-2 expression has been described by Duchnoswka et al. [22], and it has been found in about 20% of patients by Thomson et al. [23]; Schrijver et al. [24] showed that ER conversion rates were significantly higher in brain than in liver BC metastases, while PgR conversion rates were much more negligible in the central nervous system than in bone metastases. Some clinicians also reported that the loss of hormone receptors was closely associated with poorer outcomes [5]. HER2 mRNA levels were found to be increased up to five times in brain metastasis from BC tissues compared to those of primary tumors [5]. With regard to the genomic sequencing (GS) data, it has been reported that *RB1*, *ZFHX3*, *HER2*, *ATR*, *FAT1*, *ARID1A*, *ATM*, *CHEK2*, *TP53*, *BRCA1*, *CDH1*, *PTEN*, *COL6A3*, *FLT3*, *MLH1*, *BRCA2*, *MAP3K1*, *IGFN1*, *KMT2D*, *MET*, *PIK3CA* and *KMT2C* are the most frequently mutated genes in BC metastases of the central nervous system [14]. Several authors investigated the GS differences between primary and metastatic BC that has spread the brain [5]; in this regard, the overexpression of *FGFR4* and *FLT1*, combined with the downregulation of ESR1, has been reported [5]. Brain metastases and primary lesions showed similar rates of mutations of *RB1*, *PIK3CA*, *LH1*, *RB1* and *KIT*, while *TP53* was more frequently mutated in the former [5]. Based on these studies, it is possible to deduce that, as brain metastases from BC and primary tumors may exhibit different immunophenotypes and GS data, clinical oncologists should take into account the most appropriate and “personalized” therapeutic approach for these patients. For this purpose, the potential use of drugs targeting mutated genes in the BCBM, including abemaciclib, entrectinib and GDC-0084, was evaluated by a phase II clinical trial (NCT03994796).

Some studies have tried to histologically detail the microenvironment of brain metastases from BC in order to provide prognostic guidance and, perhaps, reveal new molecular targets for future therapeutic options [6]. Sambade et al. reported four histopathological biomarkers found within the breast cancer brain metastases microenvironment, namely gliosis, immune infiltrate, hemorrhage and necrosis, and assessed their associations with breast cancer subtypes along with their prognostic significance. The study demonstrated that gliosis and immune infiltration correlated with a better prognosis, while the presence of necrosis was a poor prognostic finding; in more detail, it was shown that gliosis correlated with a better prognosis in the triple-negative subtype, while the immune infiltrate conferred a better prognosis in the HER-2-enriched subtype. Specifically, the immune composition of tumor infiltrating lymphocytes (TILs), macrophages, programmed cell death protein-1 and -2 receptors (PDL-1 and -2) and the glial fibrillary acid protein (GFAP) were assessed, and it was found that the expression of PDL-1 on tumor-infiltrating lymphocytes positively correlated with overall survival [6]. When compared to a model that used purely clinical variables to assess prognosis and treatment decisions, the histological biomarkers provided a higher predictive value [6]. Therefore, including these histopathological features in pathology reports of brain metastasis from breast cancer cases could positively impact prognostic information.

In the last few years, some authors have evaluated the radiological extent of tumor necrosis in different types of metastatic tumors in the central nervous system and correlated it to the outcome of patients surgically treated by craniotomy [25]. They found that brain metastases from lung cancer (neuroendocrine and squamous cell subtypes in particular) exhibited more extensive necrosis than those from other primary tumors, including breast, genitourinary and gastrointestinal malignancies [25]. Furthermore, highly necrotic brain metastases showed poorer outcomes after craniotomy than those harboring less necrosis [25]. With respect to our study, these authors mainly focused on the radiological extent of necrosis in brain metastases from lung cancer and did not mention the histological pattern of necrosis, while our results highlight more the fact that comedonecrosis may be a morphological feature suggestive of the mammary origin of a metastatic carcinoma in the brain.

Finally, transcriptomics models capable of studying the spatial cell-to-cell communication landscape might be used in the future to better explain distinct cell morphologies and/or adaptive responses to the tumor microenvironment of metastatic neoplastic cells [26].

## 5. Conclusions

Although we are aware that the above-mentioned morphological parameters are not strictly specific to a mammary origin, we believe that they may have an important diagnostic utility in leading pathologists to suspect a possible primary breast tumor and to include GATA-3, ERs, PgRs and HER-2 in the immunohistochemical panel when dealing with a brain metastasis in their daily diagnostic practice [27,28,29,30,31]. Although, to the best of our knowledge, this is the first study that aims to select purely morphological features capable of predicting the mammary origin of a brain metastasis, in our opinion, the relatively low number of cases represents the main limitation of the present study. Furthermore, we believe that it would be very interesting to evaluate whether the morphological features described in the present paper can also be seen in brain metastases from tumors originating from other organs or whether they are specific of a mammary origin. Accordingly, the future perspective of this study is to expand our cohort of cases by also including brain metastases from non-mammary carcinomas.

## Figures and Tables

**Table 1:** Clinico-pathologic features and immunophenotypes of the cases from our series.

Cases	Age (Median Value)	Site	Solid Growth Pattern	Comedonecrosis	Glandular Differentiation	Immunophenotype
*n* = 30	51.6 y (range: 34–75)	Brain (*n* = 27) Cerebellum (*n* = 3)	Diffuse (*n* = 14) Heterogeneous(*n* = 9) Focal (*n* = 7)	Diffuse (*n* = 7) Heterogeneous (*n* = 11) Focal (*n* = 9) Absent (*n* = 3)	Heterogeneous (*n* = 8) Focal (*n* = 8) Absent (*n* = 14)	Luminal-A (*n* = 3) Luminal-B (*n* = 1) HER-2- enriched (*n* = 5) Triple-negative/basal-like (*n* = 8) NA (*n* = 13)

Abbreviations: y, years; NA, not available.

## Data Availability

All data presented in this research are available from the corresponding author upon reasonable request.
